# Massive Lumbar Disc Herniation Causing Cauda Equina Syndrome That Presents As Bladder and Bowel Dysfunction in the Absence of Lower Extremity Weakness

**DOI:** 10.7759/cureus.17952

**Published:** 2021-09-14

**Authors:** Kimitaka Nakamura, Takeshi Arizono, Akihiko Inokuchi, Takahiro Hamada, Ryuta Imamura

**Affiliations:** 1 Department of Orthopaedic Surgery, Kyushu Central Hospital of the Mutual Aid Association of Public School Teachers, Fukuoka, JPN

**Keywords:** disc hernia, cauda equine syndrome, lower extremity weakness, clinical features, bladder and bowel dysfunction, postoperative outcomes

## Abstract

The purpose of this report is to examine the features of cauda equina syndrome (CES) presenting as bladder and bowel dysfunction in the absence of lower extremity weakness. Between July 2015 and July 2016, we experienced four cases of massive LDH causing CES that presented as bladder and bowel dysfunction in the absence of lower extremity weakness. Herein, we describe the clinical features of these four patients (two males and two females) who were followed for a minimum of two years postoperatively. The mean age at the time of surgery was 46.8 years (range, 37-71 years). The disc herniation lesion was at the L4/5 level in one patient, and the L5/S1 level in three. The mean interval between the onset of CES and complete surgical decompression was 10.5 days (range, 1-18 days). Postoperative outcomes were better than poor in three of four cases, while one case had residual sphincter dysfunction. LDH causing CES is considered an indication for immediate surgical decompression; however, diagnosis of CES is likely to be delayed in atypical cases of CES that present as bladder and bowel dysfunction in the absence of lower extremity weakness. Diagnosis of CES tended to be delayed in cases without lower extremity weakness. Clinicians should recognize even sensory impairment alone of the dominant area supplied by S2-4 is an important diagnostic sign of CES in the early stage.

## Introduction

Cauda equina syndrome (CES) secondary to lumbar disc herniation (LDH) is a relatively uncommon but serious condition that may progress to paralysis and permanent bladder and bowel dysfunction [1,2]; hence, it is considered an absolute indication for surgical decompression [3,4]. The best timing of surgical decompression for CES remains controversial, but most studies recommend immediate surgical decompression when the symptoms worsen [2-6]. Clinicians must recognize CES to enable immediate operative intervention to occur; however, CES is sometimes difficult to diagnose because of the ambiguous diagnostic criteria for CES [6]. CES presents with various symptoms, but it is often accompanied by pain in the lower back and the lower extremities, motor weakness, and bladder and bowel dysfunction because of massive disc prolapse [7]. We experienced four cases of massive LDH causing CES in which the patients presented with bladder and bowel dysfunction in the absence of lower extremity weakness. In patients with bladder and bowel dysfunction who do not present with intractable pain and lower extremity weakness, diagnosis of CES may be delayed and the operative outcomes might be poor. The purpose of the present study was to examine the features of CES presenting as bladder and bowel dysfunction in the absence of lower extremity weakness to highlight the features of this rare condition.

## Case presentation

We reviewed the clinical data from four patients (two males and two females) who underwent surgical decompression for LDH causing CES that presented as bladder and bowel dysfunction in the absence of lower extremity weakness between July 2015 and July 2016. The mean patient age was 46.8 years (range, 37 to 71 years), and the mean follow-up duration was 25.8 months (range, 24 to 30 months). We examined the presenting symptoms, onset, and course of CES, magnetic resonance imaging (MRI) findings, duration from the onset of symptoms to surgical decompression, operative procedure, and postoperative outcomes. The pattern of onset of CES was classified as sudden or insidious [2]. Postoperative outcomes for CES were classified as excellent if full recovery of bladder dysfunction and the altered urethral sensation was regained within one month after surgery, good if the recovery was ultimately full but delayed, fair if recovery of bladder dysfunction was regained but the altered urethral sensation has remained, and poor if they remained incontinent.

Results

The characteristics of the four cases are summarized in Table 1.

**Table 1:** Summary of the characteristics of the four cases of cauda equine syndrome Classification of CES following the severity of sphincter dysfunction is divided into two stages: incomplete CES (CESI) according to the severity of sphincter dysfunction, which is characterized by reduced sphincter function; and CES in retention (CESR), which is characterized by complete loss of sphincter function. Postoperative outcomes for CES were classified as excellent if full recovery of bladder dysfunction and the altered urethral sensation was regained within one month after surgery, good if the recovery was ultimately full but delayed, fair if recovery of bladder dysfunction was regained but the altered urethral sensation has remained, and poor if they remained incontinent.

Case	Age(years)/Sex	Affected level	Pattern of onset	Initial symptoms	Magnetic resonance imaging findings
1	71/F	L5/S1	Sudden	Left sciatica Altered urethral sensation	Central lumbar disc herniation with central migration Lumbar spinal stenosis with degeneration
2	38/F	L5/S1	Insidious	Lower back pain Right sciatica Altered urethral sensation	Central lumbar disc herniation with distal migration
3	37/M	L4/5	Insidious	Lower back pain Right sciatica Altered perirectal sensation	Central lumbar disc herniation with distal migration
4	41/M	L5/S1	Insidious	Lower back pain Left sciatica Diminished perineal sensation Delayed micturition	Central lumbar disc herniation with distal migration

Case	Interval from symptom onset to surgery	Symptoms at the time of surgery	Classification of CES	Surgery	Postoperative outcome
1	17 days	Urinary hesitancy Anal sphincter dysfunction	CESR	Bilateral laminectomy	Fair
2	1 day	No progression of symptoms	CESR	Bilateral laminectomy	Good
3	18 days	Urinary hesitancy Loss of anal sphincter function	CESI	Micro endoscopic discectomy, followed by bilateral laminectomy	Poor
4	6 days	Pain in the perineal region Decreased desire to void Anal sphincter dysfunction	CESR	Micro endoscopic laminoplasty	Fair

**Figure 1:**
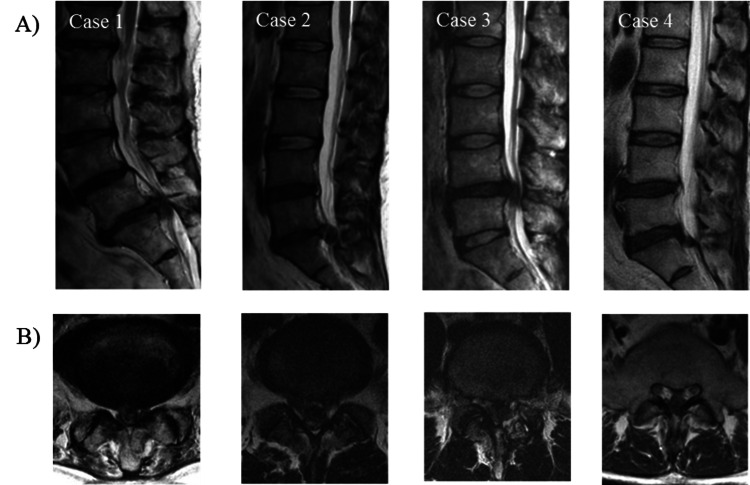
Sagittal and axial magnetic resonance imaging showing lumbar disc herniation of four cases. (A) Sagittal view. (B) Axial view.

The onset of CES was sudden in one patient and insidious in three. The initial bladder and bowel dysfunction were mild conditions such as altered urethral sensation and perirectal numbness in all patients. No patients presented with lower extremity weakness in addition to the onset of bladder and bowel dysfunction. Bladder and bowel dysfunction progressed to a severe condition such as loss of desire to void and/or urinary hesitancy in three patients. No patient had lower extremity weakness at the time of surgery. Analgesic medication achieved a certain degree of pain control in all patients, and no patient had intractable pain at the time of surgery. The mean duration from the onset of symptoms to surgical decompression was 10.5 days (range, 1 to 18 days). The level of disc herniation was L4/5 in one patient, and L5/S1 in three. MRI showed that all patients had massive central LDH, and one patient had lumbar spinal canal stenosis with degeneration (Figures 1A, 1B).

One patient (Case 3) underwent microendoscopic discectomy initially, and subsequently underwent laminectomy because of residual herniation; the other three patients underwent bilateral laminectomy and discectomy, one of which was an endoscopic procedure. Postoperative outcomes were good in one patient, fair in two, and poor in one.

Case presentation

Case 1

A 71-year-old female suddenly experienced the left lower extremity pain and altered urethral sensation after backward bending. Despite conservative treatment by a local physician and urological physician, her symptoms progressed to urinary hesitancy and anal sphincter dysfunction. She was admitted to our hospital 11 days after the initial onset of altered urethral sensation. MRI and myelography revealed a central LDH with central migration at L5/S1 and lumbar spinal stenosis with degeneration. We performed decompression 17 days after the initial onset of altered urethral sensation. The decompression was conducted via bilateral laminectomy and discectomy. Though there was a gradual improvement in urinary hesitancy and anal sphincter dysfunction at three months, the altered urethral sensation has remained at two years postoperatively.

Case 2

A 38-year-old female had chronic lower back pain and right leg pain, which was managed conservatively by a local physician. After several months, the patient experienced an acute exacerbation of the lower back pain and right leg pain. The patient noticed an altered urethral sensation one week later, and so she sought medical attention from an emergency physician. The next day, the emergency physician referred the patient to us for evaluation of a possible neurogenic bladder. At the time of presentation to our department, the lower back pain and right leg pain were relieved. On examination, there was no weakness in the lower extremities. The patient had altered urethral sensation in the absence of urinary hesitancy, and the rectal tone was normal. MRI revealed a massive central herniated disc at L5/S1 that was severely compressing the dural sac. We performed emergency decompression one day after the initial onset of altered urethral sensation. The decompression was conducted via bilateral laminectomy and discectomy. After surgery, there was a gradual improvement in the altered urethral sensation, and this was fully resolved at three months postoperatively.

Case 3

A 37-year-old male had been experiencing low back pain and right sciatica for a week and was rushed to our hospital after experiencing pain progression and altered perirectal sensation. MRI revealed a central LDH with central migration at L4/5. The initial decompression was conducted via microendoscopic discectomy. Despite the surgery, there was no improvement in symptoms before surgery. Postoperative MRI residual revealed herniation, and we strongly recommended performing a second surgical decompression. It took several days to obtain consent for surgery because he desired conservative treatment. His symptoms progressed to urinary hesitancy and loss of anal sphincter function during the period before he consented to surgery. Finally, the complete decompression was conducted via bilateral laminectomy and discectomy 18 days after the initial onset of altered perirectal sensation. Urinary hesitancy and loss of anal sphincter function were residual at three months postoperatively. Though these symptoms recovered two years after surgery, altered perirectal sensation remained.

Case 4

A 41-year-old male had chronic lower back pain, which was managed conservatively by a local physician. Though he once experienced relief of the symptom improvement, he had low back pain again, as well as left lower extremity pain, diminished perineal sensation, and delayed micturition after several months. A few days after the onset of these symptoms, he visited our hospital. MRI revealed a central LDH with central migration at L5/S1 and lumbar spinal stenosis with degeneration. We performed decompression six days after the initial onset of diminished perineal sensation and delayed micturition. At the time of surgery, decreased desire to void and anal sphincter dysfunction had appeared. The decompression was conducted via microendoscopic laminoplasty and discectomy six days after the initial onset of diminished perineal sensation and delayed micturition. Though there was a gradual improvement in decreased desire to void and anal sphincter dysfunction at three months, the diminished perineal sensation has remained at two years postoperatively.

## Discussion

CES is a neurologic disorder that can result from LDH with excessive compression of the cauda equina. The clinical features of CES include severe lower back pain, bilateral or unilateral sciatica, saddle anesthesia, motor weakness, sensory deficits, and bladder and bowel dysfunction [1,2]. The rectal or urinary disorders associated with CES vary greatly and include altered urethral sensation, loss of desire to void, poor urine stream, feeling of urinary retention, micturition by straining, perirectal numbness, and loss of rectal control. The typical onset of CES is lower back pain and numbness of the lower extremities followed by bladder and bowel dysfunction accompanied by lower extremity weakness [2,3,8]. However, the progression of CES can also vary in speed, symptoms, and severity [9,10].

Gleave and McFarlane originally reported the classification of CES following the severity of sphincter dysfunction into two stages: incomplete CES (CESI), which is characterized by reduced sphincter function; and CES in retention (CESR), which is characterized by complete loss of sphincter function [11]. Srikandarajah et al. reported that decompressive surgery within 24, 48, and 72 hours improved bladder outcomes at the initial follow-up appointment in patients with CESI, but not in those with CESR [12]. Hence, clinicians need to recognize the presence of CES promptly and perform decompression before it progresses to CESR. Sun et al. reported that the early stage of CES mainly involves bilateral peripheral nerve dysfunction, and is characterized by sensory/motor deficits in the lower extremities [13]. They also emphasized that clinicians should pay attention to the early stage CES symptoms, especially the characteristic progressive sensory/motor deficits that are sometimes followed by rectal or urinary disorders [13]. In one case (Case 1) of this study, the patient had been continued to get outpatient treatment at the clinic without being consulted with spine surgeons while having sciatica and altered urethral sensation. Since the neurological symptoms, including CES, had progressed by the time of surgery, the delay may have affected the postoperative outcome. Therefore, the clinician might not recognize that this clinical presentation was CES, which could progress to severe bladder disorder because it was only a sensory disorder without lower limb muscle weakness.

None of the present four patients in the current study presented with lower extremity weakness and intractable pain at the onset of CES, despite the absence of comorbidities that can cause sensory disturbances, such as diabetes. Several studies reported cases in which LDH causing CES in the absence of lower extremity weakness [14-16]. All cases presented large central disc protrusion at L4/5 or L5/S1, as with our cases. This finding suggests that the affected level of the massive disc herniation is an important point. The bladder and external sphincter are innervated by somatic fibers that originate in the sacral spinal cord. These fibers travel through the cauda equina and exit through the S2-4 roots. Impairment of the dominant area supplied S2-4 is mainly caused by serious compression of the dural sac at the lower level of the lumbar spine, especially at the L5/S level. Also, a central disc herniation may impinge only the sacral roots, avoiding the lumbar roots and causing neither motor weakness nor pain in the lower extremities. In addition to the absence of lower extremities weakness, bladder and bowel dysfunction was insidious in the current study. Some studies reported urodynamic studies were useful methods for the diagnosis of the neurogenic bladder dysfunction caused by LDH [17,18]. On the other hand, Inui et al. reported that the prevalence of bladder dysfunction detected by urodynamic studies in patients with lumbar spinal stenosis and LDH was much higher than that of urinary symptoms reported subjectively by the patients. Additionally, only altered urethral sensation without voiding disturbances is not likely to be detected by urodynamic studies [19]. Therefore, it is important to recognize CES only by changes in urethral sensation, even if they are not accompanied by dysuria.

In the current three out of four cases, they presented no loss of desire to void or urinary hesitancy, only altered urethral sensation. These clinical conditions were the early stage of CES described above, and the clinicians might not have been aware of the potential progression to severe bladder disorder. Moreover, symptoms such as altered urethral sensation and perineal paresthesia could have been hesitant to be reported by the patients themselves due to embarrassment. Therefore, all clinicians, not only spine surgeons, should recognize even sensory impairment alone is an important diagnostic sign of CES in the early stage and be willing to check the presence of sensory impairment without waiting for the patient to report them themselves.

## Conclusions

In conclusion, four cases of CES with bladder and bowel dysfunction in the absence of lower extremity weakness were reviewed. The atypical presentation caused a delay in the diagnosis of CES, which may have led to unfavorable outcomes. All four cases were considered to be pathological conditions in which dominant nerves of the bladder were selectively compressed and impaired. Diagnosis of CES tended to be delayed in cases without lower extremity weakness. Clinicians should recognize even sensory impairment alone of the dominant area supplied by S2-4 is an important diagnostic sign of CES in the early stage.
